# Editorials

**Published:** 1891-07

**Authors:** 


### HOMŒOPATHIC PHYSICIAN,

A MONTHLY JOURNAL OF

HOMŒOPATHIC MATERIA MEDICA AND CLINICAL MEDICINE.

"If our school ever gives up the strict inductive method of Hahnemann, we are lost, and deserve only to be mentioned as a caricature in the history of medicine."—CONSTANTINE HERING.

Vol. XI. JULY, 1891. No. 7.

### EDITORIALS.

MISCARRIAGE.—Given a case of inevitable miscarriage, what is the best method of ending it safely and as quickly as possible?

The treatment of this condition by allopathists is, as is usual with them in the treatment of all affections, very variable.

One advocates active measures for removing the membranes which may not be at first expelled; such as using the finger for their removal, or the curette, or ergot in some form. Another upholds the plan of doing nothing but tamponing and waiting for the placenta to be forced out by uterine contractions; but all have sepsis before their vision, and various harmful drugs for its prevention.

For the past nineteen years we have invariably resorted to a simple method in the treatment of this condition, and it has not failed in one case. It is simply to tie the cord and wait for the expulsion of the placenta, which usually occurs in from six to twenty-four hours.

In cases where gestation has not advanced far enough for the formation of the cord there is usually but little difficulty in treating such with the indicated remedy. It is, of course, understood that in all cases abnormal symptoms are to be treated in the same manner as symptoms in other conditions, by the simillimum.

G. H. C.

Ophthalmia neonatorum.—In a recent article on this subject, by an English ophthalmic surgeon, it is acknowledged that "a moderate estimate gives thirty per cent. of all cases of blindness as due to this disease alone." The same writer says: "I have kept a record of all children admitted into the Sheffield School for the Blind since its opening. I find that after excluding three (which were not seen by me, or for some other reason) there is up to the present a total of 116. Of these in no fewer than 46 can the cause of their blindness be traced to the disease of which we are speaking—a percentage of 39.6."

He then argues for the use of sublimate solution and silver nitrate as preventives of the affection. As these substances are in almost general use for this purpose by old-school practitioners, we fail to see the benefit arising from their use, taking his own figures as a guide.

We should like to ask whether any Hahnemannian has ever seen blindness follow ophthalmia of the new-born treated homœopathically?

If any one has had such a result, we can say from experience that he has then not followed the teaching of Hahnemann in treatment. And we are sure that the conditions in which loss of sight should occur, under genuine homœopathic treatment, must be very extraordinary indeed.

We have repeatedly had aggravated forms of this affection to treat, and in no one case has the result been other than favorable, each case always terminating with perfect vision and a clear cornea.

We have frequently restored lost vision—lost through such treatment as advised by the above-quoted writer—from nebulous cornea by following Hahnemann's teachings.

As we have often written, it is a great pity that patients of old-school practice cannot read old-school journals, for they would then be better able to judge of the results of such treatment.

In the absence of this knowledge it is incumbent upon us to enlighten them. G. H. C.

Heart Failure.—For the past several years the term heart failure has occupied a prominent place in old-school journals. The same term is now glibly used by many of the laity, more particularly since the influenza epidemic of 1889–90. Like many other terms with which our old-school friends are so ready to cover their ignorance, the flippant use of this one has come to have a meaning full of terror to many victims of drugs, as well as to others who have been fortunate enough to escape drugging.

The number of deaths charged to "heart failure" is rapidly increasing, and if there be no other apparent cause for sudden death, this affords an easy way to pacify the mourning relatives.

It is time the public should be enlightened on this subject, and assured that death never occurs without heart failure, and that preceding this there must be conditions—brought about by disease or drugs, or both—which bring about heart failure, and, consequently, death.

Formerly the poor liver had put upon it the burden of all ailments occurring, but now the heart is being burdened with more than it can bear—and who can wonder that it should fail?

